# Assessment of Epidemiological Safety in the Cosmetic Service Industry in Poland: A Cross-Sectional Questionnaire Study

**DOI:** 10.3390/ijerph18115661

**Published:** 2021-05-25

**Authors:** Anita Gębska-Kuczerowska, Izabela Kucharska, Agnieszka Segiet-Święcicka, Marcin Kuczerowski, Robert Gajda

**Affiliations:** 1 Collegium Medicum, Cardinal Stefan Wyszynski University, KazimierzaWóycickiego 1/3, 01-938 Warsaw, Poland; 2 Chief Sanitary Inspectorate, Targowa 65, 03-729 Warsaw, Poland; i.kucharska@gis.gov.pl; 3 Faculty and Department of Experimental Physiology, Medical University of Warsaw, ŻwirkiiWigury 61, 02-091 Warsaw, Poland; agnieszkasegiet@gmail.com; 4 Clinical Department of Oncological Gynecology and Obstetrics, prof. Witlold Orłowski Hospital, Czerniakowska, 231, 00-416 Warsaw, Poland; mkuczerowski@vp.pl; 5 Gajda-Med Medical Center, ul. PiotraSkargi 23/29, 06-100 Pułtusk, Poland; gajda@gajdamed.pl

**Keywords:** cosmetic service providers, epidemic safety, needlestick injury, knowledge

## Abstract

The variety of current cosmetic procedures has increased the potential risks of adverse events and infections. In a nationwide cross-sectional study (2013–2015), we assessed the aspects of infection risk in cosmetic services. An anonymous voluntary questionnaire survey was conducted among 813 employees of cosmetic establishments in Poland. The establishments were selected from a register of service providers. The survey was conducted by employees of the State Sanitary Inspectorate during an audit, and the results showed that cosmetic providers were not fully prepared for risk assessment in terms of occupational exposure or infection transmission. The majority of the respondents (84%) reportedly washed the salon tools. Some establishments did not perform any decontamination (2%) or sterilization (~13%) procedures. Occupational punctures or lacerations occurred from needles, ampoules-syringes, or razors. Most respondents had attended professional training or studied medical textbooks. Approximately 1.7% of the respondents had not updated their knowledge, and 5% gained knowledge from unauthorized sources.The project’s results impacted a variety of innovations and improvements in the field of public health. The results were used to update the national education program (2012–2017); more attention has been directed toward effective education in infection prevention, general hygiene, and post-exposure procedures. Moreover, the study’s results were grounds for the introduction of legislative modifications in the field of epidemiological safety standards for cosmetic services in Poland.

## 1. Introduction

The history of being mindful of having a perfect personal image by using makeup goes as far back as humanity’s history. The importance of appearance has had very different motivating factors over time [1]. Undoubtedly, the history of cosmetology shows that people have often prioritized their appearance over health safety concerns, and warnings about risks have often been ignored [2,3]. With the proliferation and availability of various cosmetic procedures, many of them invasive, the risk of adverse events, including infections, increases. Infectious diseases have been the main cause of occupational diseases in Poland, but the beauty industry has not been included as a major occupational area of infection risk in the register of occupational diseases [4,5]. Unfortunately, high social pressure for a beautiful image, including by the media, does not always acknowledge the health risk issues and consequences of this burden [6].Disapproval of natural beauty, denial of the aging process, and indifference to the economic implications of health complications (e.g., the issue of recording adverse events) impose other priorities, often relegating safety issues [6,7].The increasing demand for invasive cosmetic services requires safety regulations (RES AP 2008 [1] with further amendments) [8].

Several studies indicate that in medicine and cosmetology, the key to preventing infection includes an awareness of the risks and methods to prevent transmission of diseases;the application of these methods in practice includes cleaning, disinfection, and sterilization of equipment [7,8,9]. From a public health perspective, all cosmetic service providers should follow legal regulations regarding ensuring epidemiological safety during cosmetic procedures. In addition to awareness, the ability to follow the principles of asepsis and antisepsis is key to preventing infections, along with maintaining hand hygiene and correctly using personal protective equipment (PPE).

The disruption of the body’s physiological protective barrier allows the direct introduction of pathogens into the body, causing local bacterial, fungal, or viral infection and inducing a systemic response against infection [9,10,11].Cosmetic service workers, their clients, and workers in other services with proximal patient/client contact are also exposed to airborne infections, such as influenza, parainfluenza, tuberculosis, and other diseases such as the coronavirus disease (COVID-19) [12,13]. A cyclical analysis of the causes of occupational diseases in Poland from 2009–2016 showed that the most common cause of occupational disease among medical and social workers was contact with biological material and patients, including those with infectious and parasitic diseases (65%) [13]. The specificity of an individual’s occupation and the procedures performed determine the extent to which an infected employee can become a source for spreading infections.

The health and economic consequences of infections can be devastating, especially when diseases spread systemically [14,15]. In Poland, the status of transmission of some infections of public health interest, such as hepatitis C, is stable [15]. Most people with hepatitis C are infected through invasive procedures in tattoo parlors, at the hairdresser’s, during ear piercing, and other procedures, such as manicures and pedicures [15,16].

In its report, the European Centre for Disease Prevention and Control (ECDC) presented worrying statistics on the incidence of hepatitis B from 2008 to 2017 in European Union (EU) countries, including Poland [17]. This included a trend for increased chronic forms of hepatitis B until 2016 and a subsequent decline. However, in 2017, 74% of the newly reported cases of acute hepatitis B infections in Poland, Italy, and Romania were caused by hospital instrumentation. The cases were reported as medical infections but with incomplete information regarding the routes of transmission. In 2017, the completeness of acute and chronic infection data was 29% and 13%, respectively, and (in Poland) the information was obtained retrospectively by interviewing 90% of the cases. Thus, the authors pointed out the necessity of completeness of the data on the transmission routes [17]. The epidemiological trends may be influenced by other factors such as the immigration of still high-risk groups (e.g., migrants), hindering the rapid prevention of infections [18]. At the May 2017 World Health Assembly, the World Health Organization proposed evaluating the effectiveness of the health sector’s viral hepatitis elimination activities by 2030, based on a reduction in the prevalence of chronic hepatitis cases and mortality of 90% and 65%, respectively. The global efforts in the next few years will show whether the adopted targets are met and whether hepatitis viruses transmitted by blood will no longer pose a significant threat to global public health [19].

This study aimed to assess the knowledge of etiologic agents of infection among professionals in the cosmetic service industry and the behaviors that influence the risk of infections, including blood-borne diseases. The novelty of the study is the nationwide nature of the data collection and in-depth analysis of blood-borne infection transmission risk in the beauty service sector. The results of the study have been incorporated into the education program for these professionals and have been used to support further legislative initiatives in Poland.

## 2. Materials and Methods

A nationwide, voluntary interview was conducted among 824 participants who perform cosmetic services in all 16 provinces of Poland (2013–2015). The inclusion criteria for participants were an active practice and a registration on the local list of service centers. Based on the database of cosmetic entities under the State Sanitary Inspectorate’s supervision, the legal cosmetic service establishments were randomly selected for the survey (50–60 centers for each voivodeship). Subsequently, all the staff (respondents) who performed beauty procedures in the selected cosmetic service centers were invited to participate in the study. The exclusion criterion was the respondent not being registered on the local list of beauty parlors. The data were anonymized, and informed consent was obtained from the participants.

The questionnaire was prepared in association with representatives of the State Sanitary Inspectorate, the Polish institution that supervises infectious disease transmission. The questionnaire was created along with professional groups of beauty parlor employees and specialists in infectious diseases, public health, and epidemiology. Respondents were assessed regarding their knowledge of etiological agents, infection risk in cosmetology procedures, and their prevention, as well as aseptic and antiseptic procedures of salon equipment. They were also questioned regarding incidents of cuts during their work. Furthermore, employees of the State Sanitary Inspectorate randomly selected several cosmetic service providers and inspected all the rooms in these establishments, their equipment, and the means used for the decontamination procedures. The approval for the project proposal was obtained from the Steering Committee (KIK 35) of “Project HCV” [20].

### Statistical Analysis

Out of the interviewed participants, only those individuals (*n* = 813) who performed cosmetic procedures involving the disruption of tissue continuity by sharp devices were included in the statistical analysis. Descriptive methods were applied to analyze the data collected in the study. For categorical variables, the number and percentage of occurrences are presented. Fisher’s exact test or the chi-square test wasused to compare the distribution of the variables between subgroups or to test the level of significance of the relationship between two categorical variables depending on the expected size in each category. The margin of error was estimated to be 3.4%at a level of confidence of 95%.Two-sided tests were reported in all the analyses. The level of significance was set at 0.05. No data imputation methods were applied. The statistical analysis was performed using the statistical package R, version 3.4.0 (R Foundation for Statistical Computing, Vienna, Austria).

## 3. Results and Discussion

A majority of the 813 respondents(beauticians from all over Poland) were women (99.6%). Most of the respondents had higher education—42.4%; bachelor’s degree (22.7%) and master’s degree (19.7%)—were post-secondary vocational school graduates (33.7%); had secondary education (21.5%); or primary education (2.4%). The respondents’ average lengthof employment was 10.6 years (standard deviation—SD, 7.9; minimum, 0.5; maximum, 44.0; median, 8.0; interquartile range—IQR, 5.0–15.0 years). The age group with the highest number of respondents was 26–35 years (47.5%),and the overall mean age was 36.0 (SD, 9.0; median, 34.2;IQR, 24.7–37.1) years. Only respondent’s years of employment had statistical significance for the highest incidence of stabbing and cutting accidents(*p* < 0.001).

### 3.1. Major Study Findings

A majority of the respondents (64%) indicated an awareness of the possibility of transmitting all types of pathogens (viral, bacterial, and fungal infections) during cosmetic procedures. The results of our survey showed that cosmetic procedure professionalsat the end of 2015 (and the stationary training pre-tests in 2012–2017 at the national educational program) had relatively insufficient knowledge and, thus, were not fully able to assess the risks concerning occupational exposure or infection transmission to the customer (lack of expertise, 57.2%) [20].

### 3.2. Infection Risk in Cosmetology Procedures and Infection Prevention

The beauticians pointed out greater effectiveness of sterilization compared to disinfection, and most of them used sterilization proceduresand disposable equipment (Table 1). Moreover, 84% of them washed used instruments. Usually, the equipment was washed and then disinfected or sterilized, and 98% of the respondents declared using one or two of the listed methods of preventing the spread of infection (disinfection, use of disposable equipment, sterilization)in their institution. Three-quarters of the respondents replied that a combination of all these methods effectively interrupted the transmission of infection. Overall, 1% of respondents used all the processes of aseptic and antiseptic management, 1% did not use any of the methods (despite performing cosmetic procedures involving the disruption of tissue continuity), and 99% of the respondents used disinfectant fluids.

### 3.3. The Knowledge of Etiological Agents

Some beauticians were more prone to stabbing and cutting accidents during work than others (Table 2). Beauticians with knowledge of infection risk were more likely to ask clients about current/past infectious diseases than those without such knowledge and were more careful at work (Table 3 and Table 4).

Among the respondents, 36% used sterile gloves, while the majority (67%) used non-sterile diagnostic gloves (Table 5). A positive phenomenon was the significantly more frequent habit of changing gloves after a break during work (during permanent make up, 76.7%) or after a self-injury (88.2%) among respondents who gained their knowledge from medical textbooks (78%).

Presently, several cosmetic procedures, including invasive ones, have become available. Beauty procedures involve almost the entire human body and are often performed with the disruption of tissue continuity [20]. A beauty salon carries a real risk ofexposing the clients and employees to bacterial, viral, fungal, and parasitic infections during procedures [9,10,12]. The customers, staff, contaminated materials, surfaces, equipment, and devices located in the beauty salon can be sources of infection from pathogenic microorganisms. Proper adherence to aseptic and antiseptic principles, sterilization, disinfection, and cleaning processes play vital roles in infection prevention for all invasive procedures [21,22].

An overwhelming majority (for different pathogens, ranging from 96% to 99%) of respondents indicated the possibility of transmitting viral, bacterial, and fungal infections during cosmetic procedures. Only 65% of the participants perceived this risk of infection by parasites, and 64% stated the chance of infection by all the types of pathogens (Table 6). Conclusions from medical settings pointed that incomplete knowledge of infection etiology in their clinics may translate into incorrect diagnosis of diseases, resulting in missed or flawed decisions, leading to the risk of transmission of infections [23]. In Poland, during our research, infections were the leading cause of occupational diseases (infectious and parasitic) in medical settings [13]. This group of medical professionals—despite having the appropriate educational background, being better equipped to diagnose the diseases, and being more knowledgeable about the relevant etiology and precautions—stated that it is not always possible to avoid infection transmission.According to the above-mentioned report on occupational diseases in Poland between 2008–2016, the most common occupational infectious diseases were caused by the following pathogens: hepatitis C virus, hepatitis B virus, *Mycobacterium tuberculosis*, *Sarcopteshominis*, *Sarcoptesscabiei*, influenza viruses, *Bordetella pertussis*, *Pseudomonas aeruginosa*, and human papilloma virus. With the advent of newer techniques and more invasive cosmetic procedures, the risk of transmission of infections, with local and systemic health consequences, for beauticians and their clients is growing, with an increasing impact on public health [9,10,12].

The risk of infection is affected by multiple factors, such as the quality of equipment and instruments used during invasive cosmetic procedures; correct application of processes of cleaning, disinfection, and sterilization; waste management at the place of their production, disposal of infectious waste; maintaining the cold chain for preparations requiring proper handling; hand hygiene; division of zones into clean and dirty; and correct sanitary infrastructure [24]. Although in antiseptic management, the use of single-use materials and equipment is preferred, especially in procedures involving the disruption of skin and mucous membrane continuity, sometimes reusable tools and equipment are unavoidable [25]. However, their safe use depends on proper preparation, that is, undergoing decontamination procedures (washing, disinfection, and sterilization) (Table 6).

The use of single-use or sterile multiple-use equipment should be the gold standard in all procedures involving the disruption of tissue continuity. However, numerous publications have highlighted that the use of single-use equipment does not guarantee safety from infections unless proper storage and handling conditions are maintained [22,23,24,25]. Similar to that in medical settings, the risk of infection can be high when reusable equipment is used for cosmetic procedures.

The respondents pointed out the greater effectiveness of sterilization compared to disinfection against the pathogens listed. The majority of respondents also used sterilization and disposable equipment, as they considered these as the most important tools for interrupting transmission of infections in the beauty salon. Furthermore, 84% of the respondents saw the necessity to wash the instruments used in the salon. Studies have shown that the elimination of biofilm (deposits) on reusable instruments increases the effectiveness of both disinfection and sterilization of instruments used in the healthcare sector [26,27].

Most cosmetics employees were trained on the principles of sterilization and disinfection. However, recognizing differences in both decontamination methods alone does not guarantee the correctness of their performance. The control question about the necessity of hand sterilization was answered incorrectly by a minority of respondents. These and other issues regarding detailed procedures were pointed out by the State Sanitary Inspectorate employees who carried out on-site inspections at the cosmetic establishments; for example, proper concentration and expiration date of disinfectants, technique, or in the case of outsourcing, observance of expiration dates, and hermeticity of equipment packages.

Everyone has the right to privacy and protection of their health information. Therefore, every employee and client should be treated as a potentially infected person, and standard procedures should be applied based on general rules, e.g., hand hygiene [28,29]. For infection prevention and health safety of clients and employees, it is important to assess the procedure’s risk (Labor Code-law). Considering these data, it appears that the emphasis on protection has been shifted more to the clients than to the cosmetology professionals. Exposure to potentially infectious biological material is high in the medical profession [30,31], and knowledge of post-exposure procedures minimizes risk [32]. Recording and monitoring of adverse events such as punctures are standard in healthcare services [33,34,35]. The risk evidence of healthcare-associated infections (HAIs) is a motivation for quality and safety improvement [36]; however, lack of evidence on this in the beautician service sector does not mean that the problem does not exist.

Our study results indicate a high potential risk of occupational infections for beauticians due to nicks and injuries during the performance of cosmetic procedures. A significantly higher percentage of respondents had experienced injuries used disposable needles (94%), pre-filled ampoules/syringes (38%), and razors (20%) compared to respondents who had not used sharp equipment. In light of these results and the rapid increase in invasive cosmetic procedures with sharp instruments, it is worth considering better protection for cosmetic service workers, similar to that for medical personnel. Our study’s results also confirmed that knowledge about the risk is a factor in initiating positive changes to limiting this risk. Respondents who participated in training on post-exposure procedures (after contact with potentially infectious biological material) were more likely to cautiously undertake their professional activities (Table 3 and Table 4).

Despite the different deficit areas in beauticians’ awareness, approximately 93% of the respondents confirmed that they expanded their professional knowledge with information on invasive procedures from medical textbooks. Approximately 1.7% of the respondents did not update their professional knowledge, and 5% gained knowledge only from unauthorized sources (the internet, other people, leaflets). Meanwhile, many authors and supporters of the idea of lifelong learning drew attention to the need to improve knowledge and the constant need for updating, as a general principle of service safety [7,8]. Since our study’s aim was to analyze the educational needs of selected professional groups, the initial results of the analysis were used to prepare rich educational material on an e-learning platform [20]. This training, on a nationwide scale, washeld using educational methods such as stationary classes and e-learning. All forms of training were concluded with an examination and certification; the educational program was developed by experts and with the participation of the Chief Sanitary Inspectorate’s office. The national education project ran until 2017 and was funded by Swiss Contribution and the Ministry of Health (KIK35) [37].

The findings of our study and additional data reports from audits and knowledge on scope of infection risks in cosmetic salons were the inspiration for the further amendment of the legislation by the State Sanitary Inspectorate.The new legal regulations for this services sector include specifying the sanitary requirements. During public consultation of the legislative proposals for changes in this area (in August 2019), there were objections by the service providers that tightening the regulations will limit the cosmetic procedures market by increasing the costs of the procedures [38].

The outbreak of the COVID-19 pandemic at the beginning of 2020 in Poland enhanced the awareness of people on the facts that simple approaches and known procedures/rules (for example, hand and equipment hygiene, use of masks and PPE to control the routes of infection transmission, avoiding risky behaviors, and vaccination need), are necessary in the fight against communicable diseases and that prevention procedures are very important in disrupting the spread of many infections agents.This is particularly true for services involving the disruption of tissue continuity and where contact distance with the client is relatively short. The global outbreak of COVID-19 emphasized that even if the general rules to avoid spreading of infections are known, one must also be prepared against unknown infectious agents and their new pathogenicity. Moreover, in 2020, in a magazine for cosmetic professionals, an article titled “What has COVID-19 taught us? Sustainability and not just an emphasis on profit”drew attention to the need for ecological solutions for disposable packaging and refilling stations for cosmetics [39]. Looking optimistically at the article title, but critically at its content, it seems that confronting an infectious disease during the pandemic has maybe highlighted the lack of knowledge regarding risk of infection in the cosmetic care industry.This is aside from the fact that health safety is a broad concept and probably neither understood nor respected by all people similarly and equally. For example, there are new queries about whether the proposed reusable packaging will be personalized, and whether the cosmetic formulations used from bulk containers will be safe from microbes [40]. New developments often bring further questions and expectations, and with the existing issues unresolved, this may not help to create an atmosphere of trust among aware customers. There is probably a misunderstanding of the basic principle of global profit. True global solutions should consider both the guarantee of microbiological safety during high-risk procedures and respect for the ecological approach (waste disposal vs. re-use of waste). In Poland, a “positive effect” of the COVID-19 pandemic is the widely respected PPE use and decontamination requirements that are applied to most services. In addition, increased public awareness of the fact that compliance with the law and sanitary regime can stop the transmission of infections became evident through infection statistics. The dynamics of infections such as blood-borne infections are not always as high as in COVID-19 infections, with clinical signs and symptoms taking months, sometimes years, to appear. Hence, linking them to risk is often difficult, as also is attributing responsibility for the resulting infection. This was mentioned by the authors of the recent ECDC report on blood-borne diseases [17]. In 2017, most new cases of acute hepatitis B reported in EU countries were related to HAIs (e.g.,they were reported as medical infections). Due to the incomplete information on the transmission routes of registered infections (71% for acute infections and 87% for chronic infections), determining the actual source and route of transmission is difficult. The issue of HAIs also highlights the unpredictable risks due to the new features of old (drug resistance) and new pathogens (lack of recommended treatment). It is worth emphasizing that this risk appears in relation to invasive procedures in non-medical servicesas well, and that the only way to reduce the risk is by maintaining a sanitary regime.

People performing cosmetic services and their clients are exposed to pathogenic microorganisms, which may spread by various routes, including blood. Transmission of harmful pathogens can occur directly, from the infected person (patient, carrier) to a susceptible person, such as staff and clients, or indirectly, when biological agents in the beauty salon are transmitted through the hands of employees or contact with a contaminated environment (e.g., equipment, surfaces). Having established protocols, spreading awareness on these, and ensuring their implementation by the staff form the basis for the safe implementation of services. The source of infection can be anyone; the client or the service provider [33,34,35].

In Poland, the authors of the Supreme Chamber of Control in May 2018 indicated that medical and non-medical personnel’s failure to observe the principles of hand hygiene with due diligence significantly affected the higher incidence of infections [36]. Based on the analysis of the results obtained from our study, it can be concluded that not all respondents followed the principles of hand hygiene according to the guidelines.

The only limitations of the study resulted from the cross-sectional study design, the assessment of arbitrarily selected risk, and non-inclusion of unregistered beauticians. Nonetheless, our study may provide a starting point for further in-depth analyses of other types of surveys and other risk variables for transmission of infections in the beauty services sector.

Detailed analyses of the study in this professional group informed the Swiss Contribution education program’s update from 2012 to 2017.

## 4. Conclusions

In conclusion, this study identified educational areas relevant to the beauticians’ self- and client-protection that would enable the reduction of infection transmission risk while working. The cause of adverse health events in beauty salon customers should be attributed to insufficient knowledge of service providers about the risk of infections and their prevention. The introduction of legislative changes, sanctioning legislation, and enforcing compliance with sanitary standards is in the best interest of customers and employees of the cosmetic services sector and the entire beauty industry. Both the economic “savings” (based on partial assessment of benefits and costs) in non-medical services sector and performing of procedures involving disruption of tissue continuity with sub-standard protocols, as well as the lack of adherence to the sanitary regime, increase the risk of infections. Effective infection prevention education should be mandated, either as part of the diploma or for the continuing education, of cosmetology service workers who perform procedures with a breach of tissue continuity. Hand hygiene and post-exposure procedures should be constant and repeated elements of education at each stage. Vocational education programs should be updated based on substantive and methodical issues tailored to the resources and knowledge deficits of program addressees. More invasive procedures in the beauty service sector should adhere to aseptic rules as a standard.The project results have contributed to several public health innovations and improvements in Poland.

## Figures and Tables

**Table 1:** Equipment, disinfection, and sterilization—detailed results.

Variable	Category	Total *N* (%)
Application of disposable equipment, disinfection, and sterilization	I use disposable equipment + disinfect + sterilize tools	10 (1.2)
	I apply one or two of the following methods: disposable equipment, disinfection, and sterilization	795 (97.8)
	I do not use disposable equipment + do not disinfect + do not sterilize	8 (1.0)
What type of equipment do you use?	Autoclave	263 (32.3)
	Sterilizer	205 (25.2)
	Ultrasonic washer	222 (27.3)
Who performs the disinfection of the equipment used in your parlor?	I disinfect the tools	774 (95.2)
	I have an agreement with an external entity	47 (5.8)
	Disinfection is not performed	16 (2.0)
Who sterilizes the equipment used in your parlor?	I sterilize the tools	397 (48.8)
	I have an agreement with an external entity	301 (37.0)
	Sterilization is not performed	107 (13.2)
Do you use disinfecting fluids?	Yes	806 (99.1)

**Table 2:** Single-use sharp equipment and injuries while working with clients.

Variable	Category	Total	Injuries (Stabbing)		*p*-Value
			No	Yes	
Needles	no	213 (26.7%)	195 (30.3%)	8 (5.6%)	<0.001
	yes	586 (73.3%)	448 (69.7%)	136 (94.4%)	
Razors	no	695 (88.6%)	570 (90.5%)	114 (79.7%)	0.001
	yes	89 (11.4%)	60 (9.5%)	29 (20.3%)	
Pre-filled ampoule-syringes	no	623 (79.6%)	524 (83.3%)	89 (62.2%)	<0.001
	yes	160 (20.4%)	105 (16.7%)	54 (37.8%)	

**Table 3:** Awareness and the risk of infections among beauticians from clients’ information about current/past infectious diseases (for example, viral diseases) and needle stick injuries.

Variable		Category	Needle Stick Injuries		*p*-Value
			No	Yes	
Information from the client	HBV *	no	115 (85.2%)	20 (14.8%)	0.09
		yes	358 (78.3%)	99 (21.7%)	
	HCV **	no	100 (82.6%)	21 (17.4%)	0.52
		yes	378 (79.4%)	98 (20.6%)	

* Hepatitis B virus; ** Hepatitis C virus.

**Table 4:** Awareness and the risk of infections among beauticians followingparticipation in training on post-exposure procedures and from clients’ information about current/past infectious diseases (for example, viral diseases).

Variable	Category	Question about Infectious Diseases		*p*-Value
		No	Yes	
Knowledge aboutpost-exposure procedures	no	124 (35.7%)	223 (64.3%)	<0.001
	yes	89 (19.5%)	367 (80.5%)	

**Table 5:** Procedures, sharp equipment, and source of knowledge: safety procedures employed.

**Variable**	**Category**	**Total**	**Diagnostic Gloves**		***p*** **-Value**
			**No**	**Yes**	
Sterile gloves	no	505 (63.7%)	2 (5.3%)	503 (66.9%)	<0.001
	yes	288 (36.3%)	36 (94.7%)	249 (33.1%)	
			Changed gloveseach time after touching objects not directly related to the performed procedure		
			No.	Yes	
Medical textbooks	no	200 (25.0%)	57 (39.0%)	141 (21.7%)	<0.001
	yes	601 (75.0%)	89 (61.0%)	508 (78.3%)	
			Gloves changed		
			No.	yes	
Injuries (Stabbing)	no	659 (82.0%)	3 (100.0%)	19 (11.8%)	0.002
	yes	145 (18.0%)	0 (0.0%)	142 (88.2%)	
			Changed gloves each time after a procedure break		
			No	yes	
Needle cartridges’ (permanent make up)	no	66 (26.1%)	13 (48.1%)	52 (23.3%)	0.01
	yes	187 (73.9%)	14 (51.9%)	171 (76.7%)	
			Sterilization		
			No	Yes	
Cutters	no	405 (53.3%)	316 (51.6%)	86 (61.4%)	0.04
	yes	355 (46.7%)	296 (48.4%)	54 (38.6%)	
			Sterilization		
Files	no	128 (16.3%)	92 (14.6%)	33 (23.2%)	0.02
	yes	656 (83.7%)	539 (85.4%)	109 (76.8%)	

**Table 6:** Assessment of knowledge of participants regarding risks concerning occupational exposure and infection transmission.

Variable	Category	Total *N* (%)
Identify the infections that can be transmitted during cosmetic procedures	Viral	777 (95.6)
	Bacterial	801 (98.5)
	Fungal	801 (98.5)
	Parasitic	529 (65.1)
	All the pathogens listed above	519 (63.8)
Does the sterilization process totally destroy?	Viruses	801 (98.5)
	Bacteria	811 (99.8)
	Fungi	802 (98.6)
	Parasites	672 (82.7)
	All the pathogens listed above	661 (81.3)
Does the disinfection process destroy?	Viruses	682 (83.9)
	Bacteria	739 (90.9)
	Fungi	722 (88.8)
	Parasites	472 (58.1)
	All the pathogens listed above	411 (50.6)
Identify the methods that prevent the transmission of infection, e.g., viral infection	Sterilization	807 (99.3)
	Disinfection	715 (87.9)
	Using disposable equipment	805 (99.0)
	Hand hygiene	794 (97.7)
	Washing surfaces	769 (94.6)
	Washing tools	680 (83.6)
	All the procedures listed above	616 (75.8)
Have you ever received training on any of these topics?	Prophylaxis of blood-borne infections	405 (49.8)
	The rules of disinfection and sterilization	623 (76.6)
	Post-exposure procedure	460 (56.6)
	Yes, I have been trained in all the topics listed	348 (42.8)
What have been your main sources of information on blood-borne infections, disinfection, sterilization, and post-exposure procedures?	From other people performing cosmetic procedures	406 (49.9)
	Internet	638 (78.5)
	Medical textbooks	599 (73.7)
	Leaflets and social campaigns	514 (63.2)
	Courses for beauticians	673 (82.8)
	Summary: courses for beauticians or medical textbooks	757 (93.1)
	Summary: Other people, internet, and leaflets	42 (5.2)
	Summary: I have not broadened my knowledge	14 (1.7)
Do you ask your clients about past or current viral infections? (YES response)		589 (72.4)

## Data Availability

The corresponding author had full access to the anonymized database of the study and had the final responsibility regarding the decision to submit for publication. All authors had access to the selected data. Additional access to the data can be provided upon reasonable request.

## References

[1] Parish L.C., Crissey J.T. (1988). Cosmetics:A historical review. Clin. Dermatol..

[2] Epstein S.S., Fitzgerald R. (2009). Toxic Beauty: How Cosmetics and Personal Care Products Endanger YourHealth … and What You Can Do about It.

[3] Alnuqaydan A.M., Sanderson B.J. (2016). Toxicity and genotoxicity of beauty products on human skin cells in vitro. J. Clin. Toxicol..

[4] Szeszenia-Dąbrowska N. (2010). Choroby Zawodowe w Polsce w 2010 roku. (Occupational Diseases in Poland in 2010).

[5] Świątkowska B., Hanke W., Szeszenia-Dąbrowska N. (2020). Choroby_Zawodowe_w_Polsce_w_2019_roku, (Occupational Diseases in Poland in 2019).

[6] Brown A., Knight T. (2015). Shifts in media images of women appearance and social status from 1960 to 2010:A content analysis ofbeauty advertisements in two Australian magazines. J. Aging Stud..

[7] Baumgartner A., Gautsch S. (2011). Hygienic-microbiological quality of tattoo-and permanent make-up colours. J. Verbr. Lebensm..

[8] Piccinini P., Pakalin S., Contor L., Bianchi I., Senaldi C. Safety of Tattoos and Permanent Make-Up (Final Report).Publications Office of the European Union. https://ec.europa.eu/jrc/en/publication/eur-scientific-and-technical-research-reports/safety-tattoos-and-permanent-make-final-report.

[9] Jaśkiewicz J., Jurzak M., Marek D., Goździalska A. (2016). Zagrożenia Mikrobiologiczne w Gabinetach Kosmetycznych. Zakażenia.

[10] Mikucka A., Budzyńska A., Gospodarek E. (2013). Mikrobiologia w Kosmetologii (Microbiology in Cosmetology).

[11] Hryniewicz H.J. (2006). Profilaktyka poekspozycyjna zakażeń HBV, HCV, HIV u personelu medycznego. Med. Dypl..

[12] Kukułowicz A. (2016). Higieniczne aspekty usług kosmetycznych (Hygiene aspects of cosmetic services). Environ. Med..

[13] Świątkowska B., Hanke W. (2018). Occupational diseases among healthcare and social workers in 2009–2016. Med. Pr..

[14] O’Malley E.M., Scott R.D., Gayle J., Dekutoski J., Foltzer M., Lundstrom T.S., Welbel S., Chiarello L.A., Panlilio A.L. (2007). Costs of management of occupational exposures to blood and bodyfluids. Infect. Control Hosp. Epidemiol..

[15] Zakrzewska K., Stępień M., Rosińska M. (2019). Hepatitis C in Poland in 2017. Przegl. Epidemiol..

[16] Stępień M., Rosińska M. (2015). Ogniska wirusowego zapalenia wątroby typuC w Polsce w latach 2003–2013. Procedury medyczne najczęstszą drogą przenoszenia zakażeń HCV (Hepatitis C outbreaks in Polandin 2003–2013. Medical procedures as a dominant route of HCV transmission). Przegl. Epidemiol..

[17] European Centre for Disease Preventionand Control Hepatitis B—Annual Epidemiological Report for 2017; ECDC. https://www.ecdc.europa.eu/en/publications-data/hepatitis-b-annual-epidemiological-report-2017.

[18] European Centrefor Disease Prevention and Control Epidemiological Assessment of Hepatitis B and C among Migrants in the EU/EEA. https://www.ecdc.europa.eu/sites/default/files/media/en/publications/Publications/epidemiological-assessment-hepatitis-B-and-C-among-migrants-EU-EEA.pdf.

[19] World Health Organization Global Hepatitis Report 2017. https://apps.who.int/iris/bitstream/handle/10665/255016/9789241565455-eng.pdf?sequence=1.

[20] Projekt HCV “jestem świadom” (Project Prevention of HCV Infections” I am aware”). http://www.hcv.pzh.gov.pl/Page/projekt-5-1/realizatorzy-projektu-5.

[21] World Health Organization, PanAmerican Health Organization (2016). Decontamination and Reprocessing of Medical Devices for Healthcare Facilities.

[22] Gartrell B., White B. (2021). Surviving clinical errors in practice. N. Z. Vet. J..

[23] Ogunsola F.T., Mehtar S. (2020). Challenges regarding the control of environmental sources of contamination in healthcare settingsin low-and middle-income countries—A narrative review. Antimicrob. Resist. Infect. Control.

[24] United States Department of Labor Decontamination. https://www.osha.gov/hazardous-waste/decontamination.

[25] Pérez-Ayala M., Oliver P., Cantalejo F.R. (2013). Prevalence of bacterial contamination of glucose test strips in individuals single-use packets versus multi-use vials. J. Diabetes Sci. Technol..

[26] Evangelista S.S., Guimaraes N.R., Garcia N.B., Santos S.G.D., Oliveira A.C. (2020). Effectiveness of manual versus automated cleaningon staphylococcus epidermidis biofilm removal from the surface of surgical instruments. Am. J. Infect. Control.

[27] Association of Peri Operative Registered Nurses (2016). Guideline summary: Processing flexible endoscopes. AORNJ.

[28] Bulanda M., Wójkowska-Mach J. (2016). Zakażenia Szpitalne w Jednostkach Opieki Zdrowotnej (Nosocomial Infections i n Healthcare Settings).

[29] Rozporządzenie Ministra Zdrowia z dnia 22 kwietnia 2005r w Sprawie Szkodliwych Czynników Biologicznych dla Zdrowia w Środowisku Pracy Oraz Ochrony Zdrowia Pracowników Zawodow Narażonych na te Czynniki (Dz.U. 2005, Nr 81,poz. 716 z późn. zm.). http://isap.sejm.gov.pl/isap.nsf/DocDetails.xsp?id=WDU20050810716.

[30] Yunihastuti E., Ratih D.M., Aisyah M.R., Hidayah A.J., Widhani A., Sulaiman A.S., Karjadi T.H., Soejono C.H. (2020). Needlestick andsharps injuries in an Indonesian Tertiary Teaching Hospital from 2014 to 2017:A cohort study. BMJ Open.

[31] Treviño H., Romero Arenas M.A. (2020). Systematic review of blood-borne pathogen exposure rates among medical students. J. Surg. Res..

[32] Varghese G.M., Abraham O.C., Mathai D. (2003). Post-exposure prophylaxis for bloodborne viral infections in health careworkers. Postgrad. Med. J..

[33] Bber-Gheek B., Fleischer M. (2006). Podstawy Pielęgniarstwa Epidemiologicznego (Fundamentals of Epidemiological Nursing).

[34] WHO Working Group (1989). The principles of quality assurance. Qual. Assur. Health Care.

[35] Ricciardi W., Cascini F. (2020). Guidelines and safety practices for improving patient safety. Textbook of Patient Safety and Clinical Risk Management.

[36] NIK.Zakażenia w Podmiotach Leczniczych. https://www.nik.gov.pl/plik/id,16720,vp,19276.pdf.

[37] Gębska-Kuczerowska A., Rakow L., Gaber A., Kucharska I., Sujka J. Education of Medical and Non medical Professionals as a Key Element of Blood-Borne Infection Prevention. In doc. TheProjectKIK/35 “Prevention of hepatitis C virus (HCV) infections” as an example of integrated public health interventions to reduce blood-borne infections in Poland..

[38] Wiadomości Kosmetyczne Nowe Wymagania Sanitarnedla Salonów Kosmetycznych Zbyt Rygorystyczne? (New sanitary requirements for beauty salons toostrict?) (Updated 27 August 2019). https://www.wiadomoscikosmetyczne.pl/artykuly/nowe-wymagania-sanitarne-dla-salonow-kosmetycznych,56214.

[39] Wiadomości Kosmetyczne Czego Nauczył nas COVID? Zrównoważony Rozwój a nie Tylko Nacisk Na zysk (What has COVID Taught Us? Sustainable Development, not just an Emphasison Profit) (Updated 29 December 2020). https://www.wiadomoscikosmetyczne.pl/artykuly/czego-nauczyl-nas-covid-zrownowazony-rozwoj-a-nie-,66671.

[40] Akers M.J. (2016). Sterile Drug Products: Formulation, Packaging, Manufacturing and Quality.

